# Supplementary material for: Octoploids Show Enhanced Salt Tolerance through Chromosome Doubling in Switchgrass (Panicum virgatum L.)
Source: Plants (Basel). 2024 May 16;13(10):1383. doi: 10.3390/plants13101383 (PMC11124981; doi:10.3390/plants13101383)
Supplement: Supplementary file 1 [file plants-13-01383-s001.zip › plants-2985150 Supplementary figure.pdf]

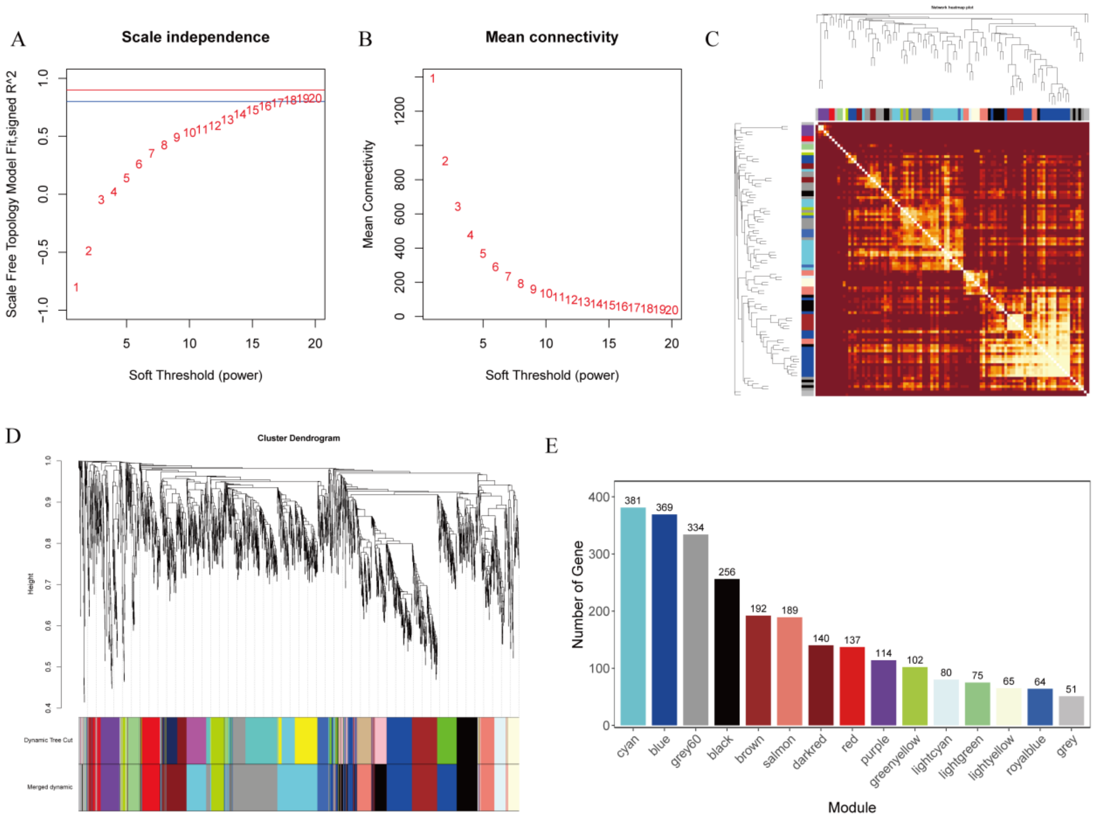

Figure S1. Determination of soft thresholdβ and the number of differentially expressed genes (DEGs) for each module. (A) The ordinate represents the index of scale free network model. The red line represents that R^2 is equal to 0.9. The blue line represents that R^2 is equal to 0.8. (B) The ordinate represents the average link degree of each's oft threshold. The abscissa represents the soft thresholdβ. (C) Module gene correlation analysis. Each row and column represents a gene, and the darker the color of each dot (white, yellow, red) represents the stronger the connectivity between the two genes corresponding to the row and column, that is, the stronger the Pearson correlation (Student's t test). (D) Cluster dendrogram and gene modules after WGCNA analysis. Different colors represent different modules. (E) Histogram of gene number of each module. The abscissa represents each module and the ordinate represents the number of genes.

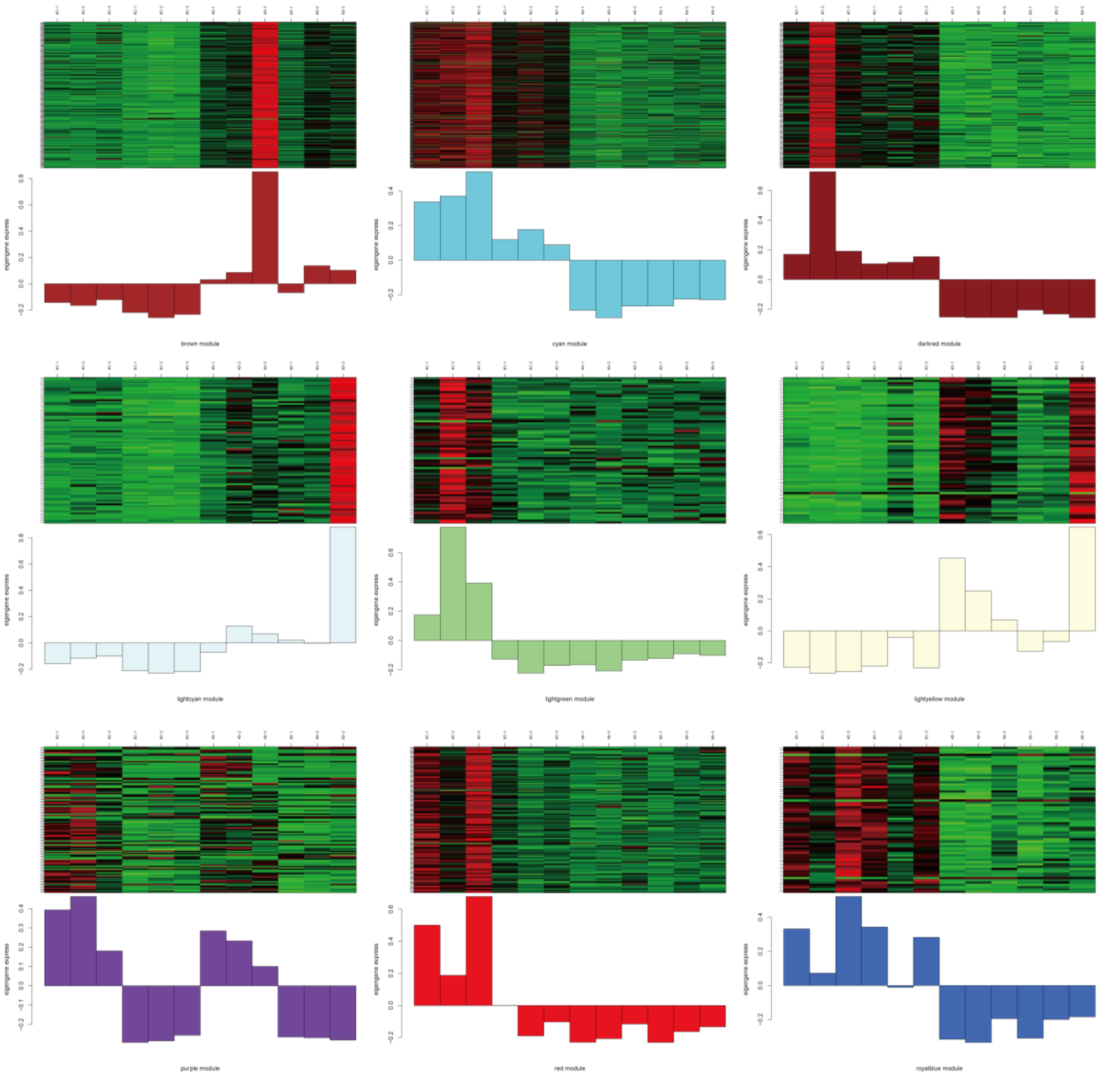

Figure S2. Heatmaps of gene expression patterns of each module. The upper part of the figure is aheat map of gene expression in different samples: red indicates up-regulation; green indicates down-regulation; the lower part of the figure is the eigengene value of the gene in different samples. The x-axis represents samples (4C, 4S, 8C and 8S) and y-axis represents the eigengene value of the gene in different samples.
