# Supplementary material for: Octoploids Show Enhanced Salt Tolerance through Chromosome Doubling in Switchgrass (Panicum virgatum L.)
Source: Plants (Basel). 2024 May 16;13(10):1383. doi: 10.3390/plants13101383 (PMC11124981; doi:10.3390/plants13101383)
Supplement: Supplementary file 1 [file plants-13-01383-s001.zip › plants-2985150 Supplementary table.pdf]

Table S1. Summary of RNA-seq data for each sample

Sample	Clean reads	Clean bases	GC content (%)	Q30 (%)
4C-1	48864640	7302935308	52.00	92.70
4C-2	52930496	7902801056	52.08	92.69
4C-3	43798104	6541085834	52.32	93.03
8C-1	53149976	7937966826	51.71	92.95
8C-2	46716486	6981044564	51.86	93.49
8C-3	46821928	6992288683	51.85	93.22
4S-1	47914230	7160991120	52.17	93.23
4S-2	41159720	6137278361	52.15	93.22
4S-3	46623136	6959197821	52.79	93.54
8S-1	48224972	7207399485	52.23	92.96
8S-2	44192820	6603142118	52.46	93.03
8S-3	49588996	7405835663	52.55	93.09

(4C: tetraploid under the control condition; 8C: octoploid under the control condition; 4S: tetraploid under salt stress; 8S: octoploid under salt stress. -1, -2, and -3 represent three replicate individuals.).

Table S2. Sequence comparison results between the sample RNA-seq data and the switchgrass reference genome

Sample	Mapped reads	Uniquely mapped reads	Multiple mapped reads
4C-1	92.16%	88.38%	3.79%
4C-2	91.09%	87.23%	3.86%
4C-3	92.42%	88.71%	3.71%
8C-1	92.40%	88.55%	3.84%
8C-2	93.06%	89.16%	3.90%
8C-3	93.17%	89.32%	3.85%
4S-1	92.86%	89.07%	3.79%
4S-2	93.20%	89.46%	3.75%
4S-3	92.76%	89.11%	3.65%
8S-1	93.26%	89.52%	3.74%
8S-2	92.83%	89.18%	3.64%
8S-3	92.55%	88.85%	3.70%

(4C: tetraploid under the control condition; 8C: octoploid under the control condition; 4S: tetraploid under salt stress; 8S: octoploid under salt stress. -1, -2, and -3 represent three replicate individuals.).
